# Supplementary material for: Modeling glioblastoma heterogeneity as a dynamic network of cell states
Source: Mol Syst Biol. 2021 Sep 16;17(9):e10105. doi: 10.15252/msb.202010105 (PMC8444284; doi:10.15252/msb.202010105)
Supplement: Supplementary file 6 — Source Data for Figure 5 [file MSB-17-e10105-s004.zip › Figure5A_sourcedata/GSEA_3017/hallmarks_stateD.GseaPreranked.1621934542601/HALLMARK_INTERFERON_GAMMA_RESPONSE.html]

Details for gene set HALLMARK\_INTERFERON\_GAMMA\_RESPONSE[GSEA]

|  || Dataset | state23017 |
| Phenotype | NoPhenotypeAvailable |
| Upregulated in class | na\_neg |
| GeneSet | HALLMARK\_INTERFERON\_GAMMA\_RESPONSE |
| Enrichment Score (ES) | -0.35892323 |
| Normalized Enrichment Score (NES) | -1.396347 |
| Nominal p-value | 0.12718786 |
| FDR q-value | 0.23475847 |
| FWER p-Value | 0.96 |
Table: GSEA Results Summary

  

Fig 1: Enrichment plot: HALLMARK\_INTERFERON\_GAMMA\_RESPONSE      
 Profile of the Running ES Score & Positions of GeneSet Members on the Rank Ordered List

  

| PROBE | GENE SYMBOL | GENE\_TITLE | RANK IN GENE LIST | RANK METRIC SCORE | RUNNING ES | CORE ENRICHMENT || 1 | BTG1 |  |  | 50 | 0.350 | -0.0035 | No |
| 2 | STAT2 |  |  | 207 | -0.254 | -0.0982 | No |
| 3 | PARP14 |  |  | 339 | -0.267 | -0.1724 | No |
| 4 | EIF2AK2 |  |  | 340 | -0.267 | -0.1457 | No |
| 5 | PSMB9 |  |  | 341 | -0.267 | -0.1190 | No |
| 6 | VAMP5 |  |  | 390 | -0.272 | -0.1288 | No |
| 7 | LY6E |  |  | 437 | -0.278 | -0.1365 | No |
| 8 | ISOC1 |  |  | 445 | -0.279 | -0.1140 | No |
| 9 | NAMPT |  |  | 450 | -0.279 | -0.0892 | No |
| 10 | ST3GAL5 |  |  | 458 | -0.280 | -0.0666 | No |
| 11 | CASP3 |  |  | 459 | -0.280 | -0.0386 | No |
| 12 | RNF213 |  |  | 533 | -0.289 | -0.0660 | No |
| 13 | AUTS2 |  |  | 590 | -0.297 | -0.0794 | No |
| 14 | BST2 |  |  | 617 | -0.303 | -0.0692 | No |
| 15 | PSMB8 |  |  | 739 | -0.328 | -0.1295 | No |
| 16 | STAT1 |  |  | 1038 | -0.438 | -0.3152 | Yes |
| 17 | ARID5B |  |  | 1095 | -0.467 | -0.3117 | Yes |
| 18 | UPP1 |  |  | 1101 | -0.469 | -0.2688 | Yes |
| 19 | IFITM2 |  |  | 1214 | -0.612 | -0.2939 | Yes |
| 20 | IFITM3 |  |  | 1279 | -0.812 | -0.2621 | Yes |
| 21 | CDKN1A |  |  | 1306 | -1.046 | -0.1777 | Yes |
| 22 | CCL2 |  |  | 1317 | -1.880 | 0.0023 | Yes |
Table: GSEA details [plain text format]

  

Fig 2: HALLMARK\_INTERFERON\_GAMMA\_RESPONSE: Random ES distribution      
 Gene set null distribution of ES for **HALLMARK\_INTERFERON\_GAMMA\_RESPONSE**
